# The effect of oxygen tension on calcium homeostasis in bovine articular chondrocytes

**DOI:** 10.1186/1749-799X-5-27

**Published:** 2010-04-26

**Authors:** Rachel White, John S Gibson

**Affiliations:** 1 Department of Veterinary Medicine, University of Cambridge, Madingley Road, Cambridge, CB3 OES, UK

## Abstract

**Background:**

Articular chondrocytes normally experience a lower O_2 _tension compared to that seen by many other tissues. This level may fall further in joint disease. Ionic homeostasis is essential for chondrocyte function but, at least in the case of H^+ ^ions, it is sensitive to changes in O_2 _levels. Ca^2+ ^homeostasis is also critical but the effect of changes in O_2 _tension has not been investigated on this parameter. Here we define the effect of hypoxia on Ca^2+ ^homeostasis in bovine articular chondrocytes.

**Methods:**

Chondrocytes from articular cartilage slices were isolated enzymatically using collagenase. Cytoplasmic Ca^2+ ^levels ([Ca^2+^]_i_) were followed fluorimetrically using Fura-2 to determine the effect of changes in O_2 _tension. The effects of ion substitution (replacing extracellular Na^+ ^with NMDG^+ ^and chelating Ca^2+ ^with EGTA) were tested. Levels of reactive oxygen species (ROS) and the mitochondrial membrane potential were measured and correlated with [Ca^2+^]_i_.

**Results:**

A reduction in O_2 _tension from 20% to 1% for 16-18 h caused [Ca^2+^]_i _to approximately double, reaching 105 ± 23 nM (p < 0.001). Ion substitutions indicated that Na^+^/Ca^2+ ^exchange activity was not inhibited at low O_2 _levels. At 1% O_2_, ROS levels fell and mitochondria depolarised. Restoring ROS levels (with an oxidant H_2_O_2_, a non-specific ROS generator Co^2+ ^or the mitochondrial complex II inhibitor antimycin A) concomitantly reduced [Ca^2+^]_i_.

**Conclusions:**

O_2 _tension exerts a significant effect on [Ca^2+^]_i_. The proposed mechanism involves ROS from mitochondria. Findings emphasise the importance of using realistic O_2 _tensions when studying the physiology and pathology of articular cartilage and the potential interactions between O_2_, ROS and Ca^2+^.

## Background

Due to the avascularity of its matrix, articular cartilage is hypoxic compared to other tissue types [1]. O_2 _tension is uncertain, but most cells probably experience 5-7% O_2 _[2]. Perhaps as a consequence, articular chondrocytes have few mitochondria and metabolism is largely anaerobic. Notwithstanding, chondrocytes consume O_2 _and are adversely affected if maintained in an anoxic environment [3,4]. Lowered O_2 _levels can occur *in vivo *in various disease conditions [2].

It is becoming increasingly evident that O_2 _tension is a critical parameter in modulating chondrocyte function [5]. At low O_2 _tension, glycolysis is inhibited, glucose uptake is reduced, and ATP and lactic acid production fall, the apparently paradoxical "negative Pasteur effect" [3]. Other responses include changes in production of growth factors, proinflammatory mediators and matrix components [5]. In other tissues, change in O_2 _tension is an important signal leading to modulation of ionic permeability and alteration of ionic homeostasis, thereby impacting upon cell function [6]. Similarly, pH homeostasis in articular chondrocytes is perturbed by alteration in O_2 _levels [7,8]. When O_2 _is reduced from 20% to 1%, the main H^+ ^efflux pathway, the Na^+^/H^+ ^exchanger [9], is inhibited leading to acidification of the cells. A reduction in reactive oxygen species (ROS) acting, via alterations in protein phosphorylation, appears to constitute the link between hypoxia and reduction in NHE activity [7].

Intracellular Ca^2+ ^levels are also critical [10]. Changes in Ca^2+ ^will affect matrix synthesis, as well as other functions. Low O_2 _tension has been shown previously to cause a rise in Ca^2+ ^in cultured embryonal chick chondrocytes, acting to slow ageing processes [11]. An interaction between O_2 _and Ca^2+ ^is therefore anticipated in articular chondrocytes but has not been described hitherto. Our overall aim therefore was to elucidate whether Ca^2+ ^levels are sensitive to O_2_. Because reduction in O_2 _tension from 20% to 1% has been shown to have important effects on pH homeostasis, we concentrated on these values for this study. Cytoplasmic Ca^2+ ^levels, ROS and the mitochondrial membrane pd were measured fluorimetrically. Results show that Ca^2+ ^levels are increased during hypoxia, with a transduction path involving mitochondrial depolarization and ROS.

## Methods

### Chondrocytes

Bovine feet from animals aged between 18 and 36 months were obtained following abattoir slaughter. Full depth hyaline cartilage shavings from the proximal metacarpophalangeal joint were taken at ambient O_2 _tension, then placed in DMEM containing penicillin (100 IU.ml^-1^), streptomycin (0.1 μg.ml^-1^) and fungizone (2.5 μg.ml^-1^) and incubated at 37°C, 5% CO_2 _for 16-18 h at 20% or 1% O_2 _whilst matrix was digested with 0.1% (w/v) collagenase type I. Isolated chondrocytes were resuspended in saline (at the required O_2 _tension) at a final dilution of 10^6 ^cells.ml^-1^. Cell viability was determined by the Trypan Blue exclusion test, at >95%. See [12] for further details.

### Solutions and chemicals

Standard saline comprised (in mM): NaCl (145), KCl (5), CaCl_2 _(2), MgSO_4 _(1), D^+ ^glucose (10) and 4-(2-hydroxyethyl)-1-piperazineethanesulfonic acid (HEPES, 10), pH 7.40 at 37°C. To investigate Ca^2+^-free conditions, CaCl_2 _was omitted and the Ca^2+ ^chelator EGTA (1 mM) added; for Na^+^-free saline, NMDG^+ ^replaced Na^+ ^- cells were prepared in standard saline and only exposed to these solutions for a few minutes. Stock solutions of digitonin, antimycin A and the fluorophores Fura-2, DCF-DA and JC-1 were dissolved in DMSO; CoCl_2 _and H_2_O_2 _were dissolved in water. Fluorophores were obtained from Calbiochem (Fura-2-AM) or Molecular Probes, Invitrogen, UK; other chemicals from Sigma-Aldrich, UK.

### Maintenance of O_2 _tension

During longer term incubations (>3 hours), cells were maintained at the correct O_2 _tension in a variable O_2_/CO_2 _incubator (Galaxy R, RS Biotech, Irvine, UK). For shorter term incubations, cells were placed in Eschweiler tonometers (Kiel, Germany) and flushed with appropriate gas mixtures using a Wösthoff gas mixing pump (Bochum, Germany). Similarly, solutions were pre-equilibrated to the required O_2 _tension in Eschweiler tonometers before being applied to cells.

### Measurement of Ca^2+^

Cytoplasmic Ca^2+ ^levels ([Ca^2+^]_i_) were measured using Fura-2 (see [12]). Cells were loaded with 5 μM fura-2-AM for 30 min at room temperature followed by 15 min at 37°C. Fluorescence was measured in a thermostatically regulated fluorimeter (F-2000 Fluorescence Spectrophotometer, Hitachi). Fura-2 was alternately excited at 340 nm and 380 nm, with emission intensity was measured at 510 nm. In most cases, the 340:380 nm fluorescence ratio (R) was converted to Ca^2+ ^values, as described previously [12]. When reagents were added to alter ROS levels, however, Ca^2+ ^levels are presented as raw R values. In these cases, exact [Ca^2+^]_i _could not be calculated because, after digitonin treatment, on exposure to the high concentrations of the reagents found extracellularly, Fura-2 was partially quenched.

### Measurement of reactive oxygen species (ROS)

Chondrocytes were loaded with DCF-DA (10 μM) at 37°C for 45 min [7]. In the presence of ROS, DCF is converted to dichlorofluorescin, resulting in a change in fluorescence. DCF was excited at 488 nm and emission intensity measured at 530 nm.

### Measurement of the mitochondrial pd

Chondrocytes were loaded with 5 μM JC-1 for 20 min at 37°C [8]. JC-1 was then excited at 490 nm and the emission intensity monitored at 525 nm (green) and 590 nm (red). The dye is sequestered inside mitochondria at negative pds. Membrane depolarization is indicated by a shift in the emission fluorescence from red to green, as dye is released into the cytosol and the formation of red fluorescent J-aggregates causing a fall in the red/green fluorescence intensity ratio.

### Statistics

Student's paired or Independent t-test were used to determine statistical significance (p < 0.05) between results. Data are given as means ± S.E.M. for n replicates, where each replicate indicates a separate individual animal.

## Results

### Effect of hypoxia on Ca^2+ ^homeostasis

**Figure 1:**
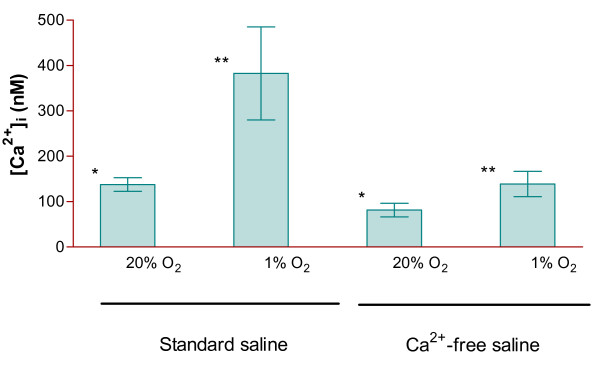
**Effect of hypoxia and extracellular Ca^2+ ^on cytoplasmic Ca^2+ ^levels in bovine articular chondroytes**. Chondrocytes were isolated with collagenase at either 20% or 1% O_2 _and maintained at these O_2 _tensions throughout (16-18 hours). Cytoplasmic Ca^2+ ^levels ([Ca^2+^]_i_) were then measured with Fura-2 in the presence (2 mM Ca^2+^) or absence (Ca^2+^-free plus 1 mM EGTA) extracellular Ca^2+^. Histograms represent means ± S.E.M., n = 9. * p < 0.02 ** p < 0.006.

**Figure 2:**
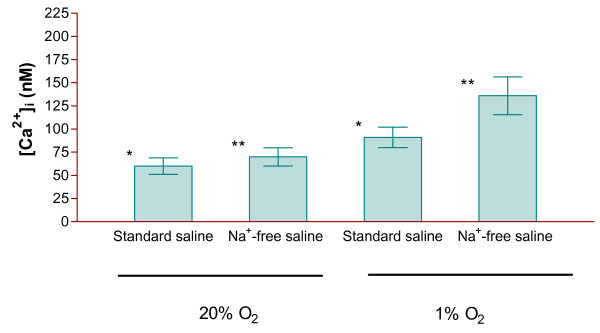
**Effect of hypoxia and extracellular Na^+ ^on cytoplasmic Ca^2+ ^levels in bovine articular chondroytes**. Methods as legend to Figure 1, except that during measurement of [Ca^2+^]_I_, chondrocytes were suspended in the presence (145 mM) or absence (Na^+ ^replaced with NMDG^+^) of extracellular Na^+^. Histograms represent means ± S.E.M., n = 9. * p < 0.05 ** p < 0.02.

Previously published reports on the effects of hypoxia on pH homeostasis in equine articular chondrocytes demonstrated effects within 3 hours when O_2 _was reduced from 20% to 1% [7]. Evidence for a similar effect was therefore tested on Ca^2+ ^levels. Bovine articular chondrocytes were isolated at 20% O_2 _and the effect of maintaining O_2 _at this level was then compared with that of reducing it to 1% O_2_. At 3 hours, [Ca^2+^]_i _was 60 ± 10 nM at 20% O_2 _compared with 62 ± 10 nM at 1% O_2 _(means ± S.E.M., n = 12; N.S. values at 1% cf 20%). At both O_2 _tensions, therefore, steady state cytoplasmic Ca levels ([Ca^2+^]_i_) remained steady at about 60 nM. We went on to study the effects of longer term hypoxia. Chondrocytes were both digested from their matrix and then maintained for 16-18 hours at either 20% or 1% O_2 _levels before measuring steady state Ca^2+ ^levels at the same O_2 _tension. At hypoxic levels, 1% O_2_, a significant elevation in steady state [Ca^2+^]_i _was observed (Figures 1 and 2), with levels approximately doubling from 55 ± 4 nM at 20% O_2 _to 105 ± 23 nM at 1% (n = 12; p < 0.001). Thus, like pH, steady state Ca^2+ ^levels in articular chondrocytes are sensitive to changes in O_2 _albeit with a slower time course.

### Hypoxia, Ca^2+ ^and ion substitutions

Ion substitution experiments were carried out to determine the source of the extra Ca^2+^. Chondrocytes were again isolated, and then maintained for 16-18 hours, at either 20% or 1% O_2 _in standard Ca^2+^- and Na^+^- containing saline. Ca^2+ ^levels were then measured in this standard saline and also following transfer to Ca^2+^-free or Na^+^-free saline (Figures 1 and 2). In Ca^2+^-free conditions (Figure 1), Ca^2+ ^was decreased at both 20% and 1% O_2_. Notwithstanding, [Ca^2+^]_i _remained higher at 1% O_2 _compared to 20% O_2_. In Na^+^-free saline, [Ca^2+^]_i _was elevated at both O_2 _tensions (Figure 2), but again remained higher at 1% O_2 _compared to 20% O_2_. In fact, the difference in Ca^2+ ^comparing cells maintained at 20% and 1% O_2 _was greater in Na^+^-free conditions.

### Interaction of reactive oxygen species and Ca^2+ ^homeostasis

Levels of reactive oxygen species (ROS) in equine articular chondrocytes decrease when O_2 _tension is reduced from 20% to 1% [7]. This finding was confirmed in the present work for bovine chondrocytes held at different O_2 _levels for 16-18 hours. ROS levels at 1% fell to 60 ± 6% (mean ± S.E.M., n = 3) of the value at 20% O_2_. Three different protocols were carried out to elevate ROS levels: treatment with the oxidant H_2_O_2 _(100 μM), the non-specific ROS generator Co^2+ ^(100 μM) or the mitochondrial complex III inhibitor antimycin A (50 μM). In each case, ROS levels recorded in treated cells incubated at 1% O_2 _were restored to those observed at 20%, (eg for Co^2+ ^levels reached 96 ± 8% values at 20%, N.S.). Using Fura-2 340 nm:380 nm emission ratio (R) as a measure of [Ca^2+^]_i_, in cells incubated at 1% but treated to raise ROS levels, it was found that R decreased by a similar amount, reaching values similar to those observed at 20%. For example, R at 1% following addition of H_2_O_2 _fell from 1.41 ± 0.001 to 1.06 ± 0.001 (n = 15). For all three protocols, therefore, at 1% O_2 _when ROS levels were restored, so was [Ca^2+^]_i_.

### Hypoxia and mitochondria

**Figure 3:**
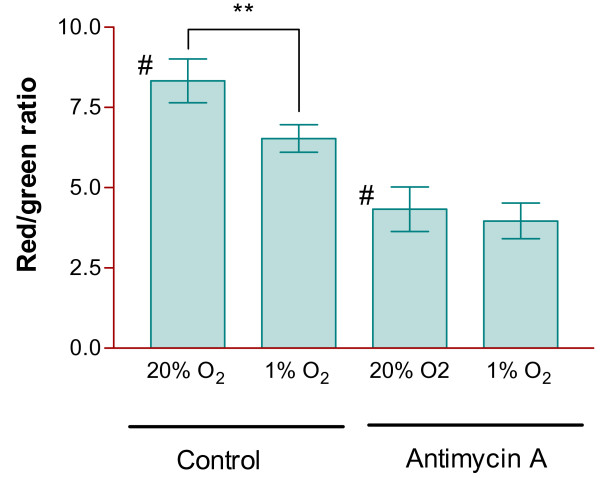
**Effect of hypoxia and antimycin A on mitochondrial membrane pd of bovine articular chondrocytes**. Chondrocytes were isolated as in Figure 1, being maintained at 20% or 1% O_2 _throughout. They were loaded with JC-1 to measure mitochondrial pd (as the red/green ratio - see Methods) in the presence or absence of antimycin A (50 μM). Histograms represent means ± SEM n = 9-11. ** p < 0.004 # < 0.002.

The effect of changes in O_2 _and treatment with antimycin A on mitochondrial pd was then investigated. Chondrocytes were isolated at 20% O_2 _and then incubated at either 20% O_2 _or 1% O_2 _for 16-18 hours prior to loading with JC-1. They were also treated with antimycin A (50 μM) at both O_2 _tensions (Figure 3). It can be seen that the red/green ratio was reduced at 1% O_2 _indicative of mitochondrial depolarization. Antimycin A, a complex III inhibitor, also caused mitochondrial depolarization at 20% O_2 _but not in cells held at 1% O_2_.

## Discussion

### The effect of O_2 _tension on steady state Ca^2+^

The present findings are the first to demonstrate an effect of changes in O_2 _tension on Ca^2+ ^homeostasis in articular chondrocytes. We show here that Ca^2+ ^homeostasis is maintained in response to shorter term (3 hours) reduction in O_2 _tension from 20% to 1%. Longer exposure to 1% O_2_, however, caused significant elevation in [Ca^2+^]_i _with levels approximately doubling, sufficient to perturb cell function. These effects were associated with both mitochondrial depolarization and a fall in levels of reactive oxygen species (ROS).

### Source of Ca^2+^

Rise in [Ca^2+^]_i _can occur through increased entry or decreased removal across the plasma membrane or from intracellular stores. It is not easy to distinguish unequivocally between these possibilities. Despite a decrease in [Ca^2+^]_i _in Ca^2+^-free saline, however, hypoxic chondrocytes still showed higher Ca^2+ ^compared to those at 20% O_2_. Thus even if increased influx across the plasma membrane was involved, other mechanisms were still able to elevate Ca^2+ ^during hypoxia. Substitution of extracellular Na^+ ^increased [Ca^2+^]_i _and exacerbated the difference at the two O_2 _tensions. This finding is consistent with elevated activity of NCE at low O_2_, perhaps in an attempt to reduce Ca^2+ ^to levels found at 20% O_2_. Since NCE activity requires a functional ATP-driven Na^+^/K^+ ^pump, it is unlikely that ATP was limiting (as shown previously [7]). In addition, because inhibition of the mitochondrial electron transport chain with antimycin A reduces [Ca^2+^]_i_, any Ca^2+ ^release from mitrochondrial stores following their hypoxia-induced depolarization, would likely to be insufficient on its own to raise [Ca^2+^]_i_. In this context, it is important to note that mitochondria in articular chondrocytes occupy a relatively small volume (1-2% cytoplasm) [13] compared to that seen in other tissues (typically 15-20%, eg liver). There is also some reduction in mitochondrial volume with depth and age [14,15]. They may also lack a functional electron transport chain [16], relying on glycolysis for metabolic energy [3]. Taken together, these findings are consistent with hypoxic release of Ca^2+ ^into the cytoplasm from intracellular non-mitochondrial stores, probably endoplasmic reticulum.

### Oxygen and chondrocyte function

As noted above, it is unlikely that articular chondrocytes require O_2 _for energy, at least directly. Nevertheless, O_2 _tension is a critical parameter in modulating chondrocyte function. Changes in O_2 _level affect ATP production [3], growth factors [17], proinflammatory mediators [18] and matrix components [19]. Dedifferentiation of chondrocytes occurs when they are maintained at abnormally high O_2_. This includes restoration of the ability to carry out oxidative phosphorylation [20]. Standard chondrocyte markers, such as collagen type II and aggrecan, are affected [19]. In effect, low O_2 _tensions (c.5%), which are normal for articular cartilage but hypoxic for other cell types, promote a chondrocyte phenotype [21-23]. In addition, however, a pathological role for O_2 _has also received considerable attention. Thus abnormally high or low O_2 _levels with concomitant alterations in levels of ROS, may be important in disease states such as osteoarthritis [24-26]. O_2 _also affects acid-base balance in articular chondrocytes [7,8]. The present findings extend the action of O_2 _to include modulation of an additional important ion, ie Ca^2+^, with low O_2 _causing intracellular [Ca^2+^] to rise. The O_2 _tension at which perturbation of Ca^2+ ^requires further definition, it being particularly important to study the likely physiological levels of between 10% and 1%.

### Calcium and chondrocyte function

Intracellular Ca^2+ ^in chondrocytes, as in other cell types, also has numerous physiological and probably pathological roles [27]. Of particular relevance to chondrocytes is the observation that perturbation of normal Ca^2+ ^levels reduces matrix synthesis [10]. It also affects both chondrocyte differentiation [28] and ageing [11]. Ca^2+ ^signalling has been implicated in a range of other chondrocyte functions including mechanotransduction [29-32], volume regulation [33-39] and response to electrical stimulation [40]. It may therefore play a critical role in how joint loading and unloading promotes cartilage health. Intracellular Ca^2+ ^elevations, for example, induce chondrogenesis via a calcineurin/NF-AT pathway [41]. Extracellular levels of Ca^2+ ^are also important in the longer term, when they too may be involved in alteration of matrix production including proteoglycan synthesis and expression of collagen [42-44] - extracellular Ca^2+ ^receptors are present. Ca^2+ ^is also implicated in the action of proinflammatory cytokines such as IL-1 and, again therefore, has received attention in the context of joint disease such as osteoarthritis [45].

### Crosstalk between oxygen, reactive oxygen species and Ca^2+^

The elevation of intracellular Ca^2+ ^at low O_2 _reported here was associated with a fall in ROS and also mitochondrial depolarization. In most cell types, though probably not articular chondrocytes, mitochondria are critical for oxdative phosphorylation and hence central to energy production. They are also involved in Ca^2+ ^regulation, acting as a sink of, or sometimes a source for, cytoplasmic Ca^2+ ^- Ca^2+ ^being released via the mitochondrial permeability transition pore (PTP) [46-48]. ROS are generated during mitochondrial respiration [49,50], as well as at other cellular sites. ROS, of course, can be harmful but have also been implicated in intracellular signalling, regulating redox sensitive enzymes and also ion channels. By these means, ROS may modulate intracellular Ca^2+^, eg acting via modulation of ryanodine receptors, IP3 receptors, Ca^2+ ^pumps and NCE [51-53]. Ca^2+ ^uptake by mitochondria may itself alter ROS generation - both reduction of ROS (through dissipation of the negative mitochondrial pd) or their elevation have been reported [54,55]. To a certain extent, the direction of change depends on tissue type and respiratory rate. Another obvious signal is represented by hypoxia-inducible factor (HIF). Stabilization of HIF1α occurs during hypoxia (eg [6,56,57]) and may affect [Ca^2+^]_i _through effects calcium channel gene expression and activity [58,59]. There is thus considerable scope for cross-talk between O_2_, ROS and Ca^2+^, together with the role of mitochondria [51,53,55] but the exact coupling in chondrocytes awaits description.

### Reactive oxygen species, mitochondria and regulation of Ca^2+^

We show here that a fall in ROS during hypoxia correlated with elevation of Ca^2+^, whilst restoration of ROS levels to those seen at 20% by three disparate reagents (H_2_O_2_, Co^2+ ^or antimycin A) all resulted in decreased Ca^2+^. Hypoxia also induced depolarization of mitochondria, indicative of a reduction in electron flow through the mitochondrial electron transport chain, and hence ROS production. Addition of antimycin A also blocks electron transport to the terminal complexes, acting at the Q_i _site of complex III to increase ROS output [8], as also observed in the present work. It is thus likely that reduced production of ROS from mitochondria is involved in the rise in Ca^2+^, as proposed for O_2_-induced changes in NHE activity and intracellular pH [8]. In the case of H^+^, however, perturbed homeostasis on change in O_2 _tension is observed rapidly, within a few minutes [60]. Effects on Ca^2+ ^appear to occur over a much longer time course, despite sharing sensitivity to ROS levels. The reason for this is not immediately apparent. It may be that Ca^2+ ^homeostasis, as a more critical modulator of chondrocyte function, is better protected than pH. Alternatively, it may be that the mechanism involves genomic effects, such as though involving HIF. In addition, a link between Ca^2+ ^and pH in chondrocytes has been shown previously, with alkalinisation causing a rise in Ca^2+ ^[61]. Since chondrocytes acidify in response to low O_2_, however, rather than increasing their pH, the hypoxia-induced rise in Ca^2+ ^cannot be secondary to changes in pH.

## Conclusion

O_2 _tension exerts a significant effect on cytoplasmic Ca^2+ ^levels of articular chondrocytes, with the proposed mechanism involving ROS from mitochondria. Results emphasise the importance of O_2 _to chondrocyte function and that of using realistic O_2 _tensions when studying the pathophysiology of articular cartilage.

## Competing interests

The authors declare that they have no competing interests.

## Authors' contributions

RW helped plan the experiments, carried them, analysed the data and helped write the manuscript; JSG planned the experiments, analysed data and prepared the manuscript.

All authors have read and approved the final manuscript.
